# A Circular Approach to Finished Tanned Leather: Regeneration by Cryogenic Technology

**DOI:** 10.3390/ma16186166

**Published:** 2023-09-11

**Authors:** Omar Salmi, Simone Gelosa, Filippo Rossi, Maurizio Masi

**Affiliations:** Dipartimento di Chimica, Materiali e Ingegneria Chimica “Giulio Natta”, Politecnico di Milano, Piazza Leonardo da Vinci 32, 20133 Milano, Italy; omar.salmi@polimi.it (O.S.); simone.gelosa@polimi.it (S.G.); filippo.rossi@polimi.it (F.R.)

**Keywords:** recycle, circular economy, cryogenic removal

## Abstract

Finished tanned leather is usually covered by a thin polymeric layer. This layer has the scope to change the morphological aspect of the last leather layer as well as improve the impermeabilization properties. Often, the finished product is refused by the final client, and tanneries must restore significant quantities of materials. Therefore, it is very important to remove this finished polymeric layer, recover the underneath tanned leather, and predispose it to a new finishing. The bonding between the polymeric film and leather is so strong that, today, only a blade shaving process can perform this separation at the expense of also removing a layer of tanned leather and consequently reducing the leather thickness. Here, a novel separation method was developed based on the significant difference in the dilation properties between the tanned hide and the polymeric film at low temperatures. The use of cryogenic fluids, in particular the direct application of liquid nitrogen, can freeze the polymeric layer below the glass transition temperature, inducing brittle behavior. The result is an easy separation without any alteration of the tanned leather layer; for a demonstration of that, some techniques were used, such as FTIR, SEM, Tensile strength evaluation, DSC, and TGA. By this last analysis, it is possible to check how a decrease of weight to 90% happened for the polymeric layer at about 400 °C against the complete blank at about 600 °C. A similar great distance of results exists in the case of tensile strength, where an average value of 34.5% is the deformation stress for blank samples, against 34.8% for processed samples. Thus, the process here developed allows the reuse of the tanned leather towards a new life in respect of the principles of the circular economy.

## 1. Introduction

Tanned leather is an important and versatile material with a lot of different applications, such as in clothing, footwear, home furniture, and car upholstery [1]. From an environmental point of view, tanned leather nobilitates a waste from the meat food chain that cannot be avoided, the animal hide [2]. Accordingly, until humans continue to eat meat, the leather market will continue. Moreover, the tanning process induces unsurpassed durability properties in the final product, justifying the success of the leather industry since the beginning of human history. However, such durability is obtained at the expense of several chemicals and at least 20 L of water per kilogram of raw hide [3]. In conclusion, when considering the traditional factors affecting the Life Cycle Assessment of the tanned leather, the impact of animal growth is significantly higher than that of the successive industrial processing of the raw hide to obtain the finished tanned leather [4,5].

Additionally, the leather processing industry is a very conservative sector. Progress was traditionally made by sustaining market demand instead of reducing environmental impacts [6]. By chance, things are rapidly changing, and the demand for more environmentally sustainable products is pushing this industry to innovate. Examples are the introduction of green tanning agents to reduce the amount of some unavoidable chemicals and plan their substitution in the near future, and last but not least, to reduce the amount of polluted waste water [7,8,9]. Focusing attention on tannery wastes, the main ones are scraps and wastewater. The formers are difficult to recycle and are mainly incinerated or landfilled [10]. These scraps represent 60% of all raw material weight [11]. Very often, they are not only the cutting edges used to select the leather core where the defects are substantially absent; however, they represent the whole piece if the client does not accept the final quality. Unfortunately, respect for the promised leather quality can be assessed only when the whole process (tanning and finishing) is complete [12,13,14]. Accordingly, the problem generated by these scraps is not “only” environmental; it also has economic relevance. If finished leather becomes waste, all the energies and chemicals used to perform that become negative voices [15].

It is then clear that it is of paramount relevance to try to recover at least the entire finished leather. The finished layer is made by a mixture of resins and dyes [16,17], and usually this layer produces visual and handy effects that are designed for the final costomer, typically a fashion firm. For this reason, a finished leather that does not pass the acceptance parameters could hardly be sold to a different customer.

A possible recovery process is based on removing the finished layer in such a way that the tanned leather underneath could be refinished again [18,19]. Unfortunately, the finished layer is strongly adherent to the tanned leather, and the only possible recycling chance requires the removal of this very adherent finished layer. This operation can be industrially performed by blade shaving it to bring the leather back to “crust” status, ready for another time for the finishing step. Unfortunately, it happened at the expense of also removing a layer of tanned leather and consequently reducing the leather thickness. In fact, the main negative aspect of that is the incomplete versatility of all kinds of leather [20]. For example, in the case of clothes made of leather, it will be difficult to remove only the polymeric layer without leaving the leather support unaltered. If it is destroyed or consumed, it will be impossible to reutilize the leather base.

In conclusion, there is space to develop a new recovery process where the delamination proceeds without the blade shaving and where the leather crust properties are preserved without the limitation of thickness [21]. The solution here proposed is cryogenic delamination. This technique is already applied for metal cleaning and in the case of paint removal [22]; however, it was never tried on leather for the finished layer separation, and the only demonstration of that in literature is a quite recent Italian patent [23]. It is based on the different dilatation properties at low temperatures that permit an efficient detachment of substances, as it is for tanned leather and polymeric finished layers.

Leather could generally resist hard conditions, preserving its structure, because the complex collagen matrix has a large quantity of strong covalent bonds [24]. Moreover, the tanning agents improve this resistance in terms of bonds between different collagen chains. On the contrary, the finished layer is a polymeric cover whose structure is different depending on the recipe used. In general, these polymers are polyurethanes and/or polyacrylates. These polymers have a glass transition temperature significantly higher than that of leather. Thus, if they are frozen below that point, their mechanical behavior shifts from elastic to brittle. Immediately, cracks appear, leading to the layer’s detachment from the leather.

As cryogenic fluids, because of their commercial availability, preference goes to liquid nitrogen, whose operating temperature approaches −200 °C. However, it is not necessary to reach these temperatures. The −80 °C of CO_2_ dry ice is also sufficient [23]. Here, it is preferably used in the form of CO_2_ snow, using the same apparatuses adopted for paint removal from metal surfaces by CO_2_ ice blasting [25].

In this work, since the process conditions are very easy to understand, attention will be focused on assessing the quality of the delaminated leather and, in particular, the finished layer removal effectivity.

## 2. Materials and Methods

### 2.1. Materials

The cryogenic delamination was carried out on different kinds of finished leather. They were three samples of commercial leather, with three kinds of colors and finished layers, for a method versatility demonstration. They were all tanned by chromium because it was the most common strategy [26]. This choice was made to discover if the new potential recycling system that was described below was suitable for ordinary leather. They had distinct characteristics, reported in Table 1:

They were analyzed using some techniques for better characterization of samples and comparison with products.

### 2.2. Cryogenic Delamination

For a lab-scale reproduction of this new method, cutted leather pieces were prepared in different dimensions, from 1 cm by × 1 cm to more. Then, they were soaked in liquid nitrogen and conserved in a Dewar vessel. Samples become completely frozen in five seconds to one minute, depending on the size. It generally happens when the usual change of nitrogen state, while liquid is in contact with something hotter, decreases temperature and liquid quantity dramatically. At this state, the leather was really contracted, and more than the collagenic crust, it was mainly the polymer layer. So, it started to be possible to delaminate the finished layer, helped by a tweezer and a slow folding action by the operator on samples. This step was carried out very carefully and patiently; in fact, there are some process issues to which to pay attention. The polymeric layer is so thin and fragile that it took a long time to eliminate it. At the same time, the collagenic crust is also (less) contracted, and if it is fast and strongly bent, the breaking risk of the biological substrate could be real. For these reasons, this is a research process to be improved by a continued nitrogen flux that will freeze and remove without changing the leather’s conformation. In any case, this is a demonstration of the different polymeric layers’ responses to the cryogenic condition. Of course, when a sample was starting to defrost a lot, it was important to restart a new batch. After that, some new samples were analyzed:

- The polymeric layer was removed to determine and characterize which compound it is.
- The resulting crust leather, to be sure of all polymeric removal and quality maintenance.
- The original sample for a better comparison.

### 2.3. Analysis

Solubility: The aim was a valuation of the polymeric layer’s solubility to analyze and characterize it in wet conditions. Different experiments were carried out on Sample 1, in vials, with 5 mm × 5 mm of this layer already removed from leather. The black one was chosen for its strong and hard layer. If there was a partial dissolution, the insoluble part was tested with other potential solvents. If there was a solvent that could solubilize the finished layer of Sample 1, this was tried with the others. After putting the pieces inside glass vials, 3 mL of solvent were added, and they were corked and agitated for 8 h at room temperature. The solvents tried were: distilled water, dimethyl sulfoxide (DMSO) (CAS: 67-68-5, Honeywell, Muskegon, MI, USA), tetrahydrofuran (THF) (CAS: 109-99-9, Sigma-Aldrich, St. Louis, MO, USA), chloroform (CAS: 67-66-3, Carlo Erba Reagents, Val de Reuil, France), acetonitrile (CAS: 75-05-8, Carlo Erba Reagents, Val de Reuil, France), ethanol (CAS: 64-17-5, Sigma-Aldrich, St. Louis, MO, USA), methyl tert-butyl ether (CAS: 1634-04-4, Sigma-Aldrich, St. Louis, MO, USA), n-hexane 99% (CAS: 110-54-3, Carlo Erba Reagents, Val de Reuil, France), dichloromethane (DCM) (CAS: 75-09-2, Sigma-Aldrich, St. Louis, MO, USA), cyclohexanone (CAS: 108-94-1, Carlo Erba Reagents, Val de Reuil, France), acetone (CAS: 67-64-1, Carlo Erba Reagents, Val de Reuil, France), toluene (CAS: 108-88-3, Fisher Scientific, Loughborough, UK), butanone (CAS: 78-93-3, Fulka Chemie, Buchs, Switzerland), methyl acetate (CAS: 79-20-9, Fisher Scientific, Loughborough, UK), ethylene glycol (CAS: 107-21-1, Carlo Erba Reagents, Val de Reuil, France), dimethyl sulphide (DMS) (CAS: 75-18-3, Sigma-Aldrich, St. Louis, MO, USA), dioxane (CAS: 123-91-1, ThermoFisher, Kandel, Germany) and dimethylformamide (DMF) (CAS: 68-12-2, Sigma-Aldrich, St. Louis, MO, USA). DMF was also tried at 100 °C for 3 days. When something was solubilized and something was not, because of the mixture of various analogous polymers with different substituents, what remained insoluble tried to be picked up and resolved in another solvent.

Spectroscopy: Fourier Transformed InfraRed (FTIR) was analyzed polymeric layers on the external and internal faces. The spectra were acquired with a Nicolet iS 10 FT-IR spectrometer equipped with an ATR Smart iTR module and a zinc selenide crystal. 64 scans were collected at 4 cm^−1^ resolution. The aim was to characterize the polymeric layer.

Microscopy: Scanning Electron Microscope (SEM) images were acquired by Zeiss Evo50 with EDS Bruker Quantax 200 6/30 probe instrumentation, used after complete freeze-drying of the samples. SEM analysis was carried out on the surface, below, and sideways of the collagen crust of Sample 1 to be sure of the polymer removal and to know the leather state before and after the treatment [27].

Tensile strength and elongation behavior: a reproduction of UNI EN ISO 3376:2020 [28], for an evaluation of mechanical properties before and after removal A universal dynamometer, Instron mod. 5900R-4505, was used, with a load cell of 5 kN and an uncertainty of 0.3 N. The samples were used in a different conformation from the normative, such as strips, for a prior study of them. For a tensile strength determination, the machine ran until the sample was broken; this is the maximum force value. At the same time, another value could be recorded: the elongation at maximum force. These values are expressed:

(1) $$T_{n}\;=\frac{F_{\max}}{w\;\times \;t}$$

where F_max_, W, t, and T_n_ are the measured forces as described above, the sample width and thickness (both expressed in mm), and the tensile strength. The sample elongation E_max_ was evaluated as follows:

(2) $$E_{\max}\;=\frac{L_{2}-L_{0}}{L_{0}}\times 100$$

L_2_ and L_0_ are the final and initial lengths of the sample, respectively.

Thermal analyses: Were carried out for the determination of different behavior under different heating conditions of polymer, crust leather, and samples [29].

- Differential Scanning Calorimetry: DSC was carried out on 5 mg of every sample that was heated and cooled between −60 and 180 °C for four times. The heating rate was 20 °C/min in a suitable sample holder. The instrument was a Mettler Toledo Polymer DSC. It allowed us to know the exact transition temperature.
- Thermal Gravimetric Analysis-Thermal Mechanical Analysis: TGA-TMA samples were heated from 30 to 900 °C at a heating rate of 10 °C/min. The instrument was a PerkinElmer STA 6000. Two analyses were coupled, and in the analysis, it was possible to see the weight decrease and the temperature paths.

## 3. Results and Discussion

### 3.1. Cryogenic Removal

**Figure 1:**
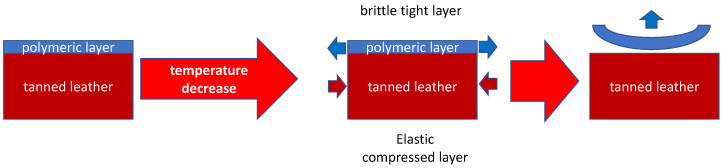
Schematic illustration of the cryogenic delamination process.

Based on the principle revealed before, a rapid freezing of finished leather creates the contraction of the polymeric layer and permits its easy separation from the underside leather. This is possible due to the different glass transition temperatures and thermal expansion coefficients between the two substances due to the high diversity of collagen and the synthetic finished layer. According to a deep analysis, as illustrated in Figure 1, the temperature changes cause a tensile compression state in tanned leather and a tensile dilatation state on the polymeric layer. This situation permits the separation as represented [23]. Another relevant point is the strong resistance of leather at these temperatures.

This is conducted by immersing the sample in liquid nitrogen [30]. Some other techniques were tried, such as cotton immersion and application on leather (to avoid possible leather damages) or nitrogen spilling on the leather surface [31]; however, the first one was the most efficient. Another possibility could be ΔT extremization by nitrogen affixation on a 40 °C leather sample to check if polymer will be removed in less time on a hotter support. Anyway, all these methods were not as useful and fast as the first.

**Figure 2:**
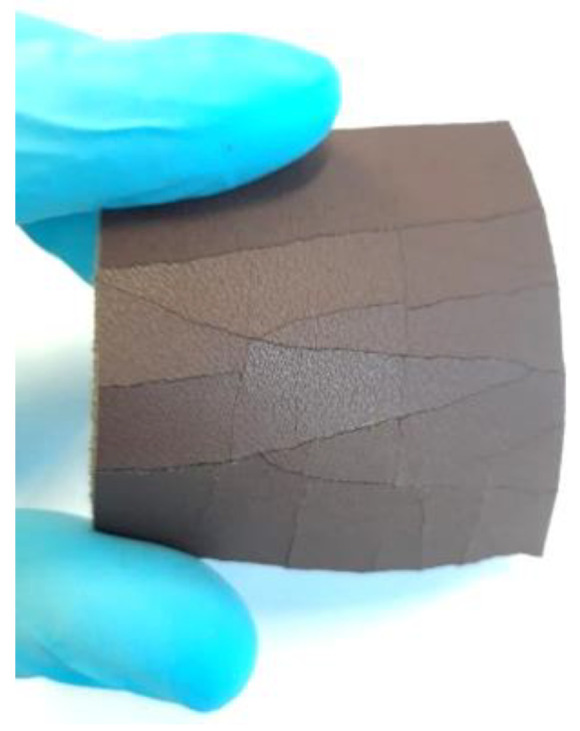
Finished layer breaks due to N_2_ liquid and slow folding.

An example of the frozen leather aspect is illustrated in Figure 2, where the cracks produced by different contractions are evident.

One of the main advantages of this idea is that the answer to thickness has remained the same in the case of leather, with only a difference of tenths of millimeters. These data demonstrated the high innovation and quality of this method, which is much better than the shaving recycling system. Moreover, in Table 1, the cryogenic polymer removal was easily observable. It allowed us to know which was the best way and to discover some interesting effects of this method.

### 3.2. Dissolution

For analyzing the chemical properties of these layers, such as molecular structure (NMR) or polymeric status (GPC), it was important to find a good solvent for them. It also permits the separation of some components with liquid chromatography and the knowledge of the mixture composition, which is very important for some properties. In fact, the contracting temperature (T_c_) depends on compounds; if these substances have a particular T_c_, it could be applied to better temperature control for cryogenic remotion.

Common solvents tried and used during the finishing step in tanning industries [32] are cyclohexanone, acetone, butanone, methyl acetate, ethylene glycol, and dioxane. Ethanol was used to represent alcohols commonly used in the same field, as said before. A common diluent was tried to be applied, normally used in the finishing polymer mixture in the tanning industry, n-hexane. Methyl tert-butyl ether was tried to represent ethers commonly used in the same field, as said before. Solvents, normally used in deuterated form for nuclear magnetic resonance (NMR) analysis to know the exact structures, were tried: distilled water, DMSO, DCM, and chloroform [33]. THF, normally used in Gel Permeation Chromatography (GPC) analysis to know and characterize different polymers, was tried. Acetonitrile, generally used in High Permeation Liquid Chromatography (HPLC), was tried. Toluene, to check the non-polar solvent dissolution activity, was tried. DMS, to know the polymers’ behavior with useful or reactive substances [34], was tried, and finally DMF, generally used for polyurethane dissolution [35]. All these compounds did not completely dissolve the polymeric layer, as could be seen in Table 2.

**Figure 3:**
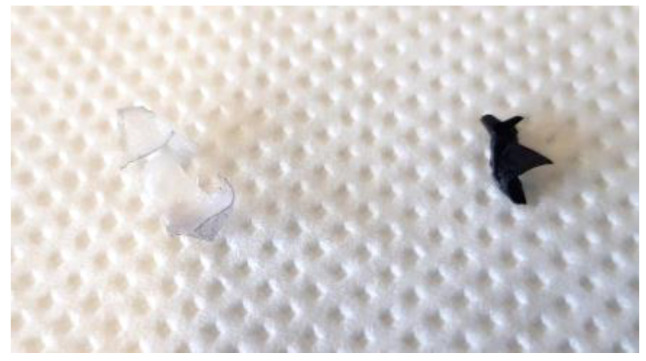
Separation of polymeric finished layers.

DMSO and DMF were the only solvents that could dissolve certainly one layer (the colored) and only in two samples (not in sample 3). After that, some lipophilic and hydrophilic common solvents were tried to check which direction must be followed. However, again, in these cases, there were not exhaustive results. It could be possible that PUs were particularly substituted and functionalized; therefore, they were not reactive such as the simplest and normal forms [36]. The polymer separation seen in Figure 3, by cyclohexanone and chloroform, permitted better analysis of the uncolored layer without the presence of the colored one. Furthermore, with the use of these solvents, samples were prepared for other analyses. The uncolored finished layer was tested with mainly solvents and seemed unactive. They were used because, if the uncolored part was dissolved, it would become undetectable on the colored finished layer of sample 1.

After this first assay, it was decided to analyze samples in the solid state due to their poor solubility.

### 3.3. FTIR

**Figure 4:**
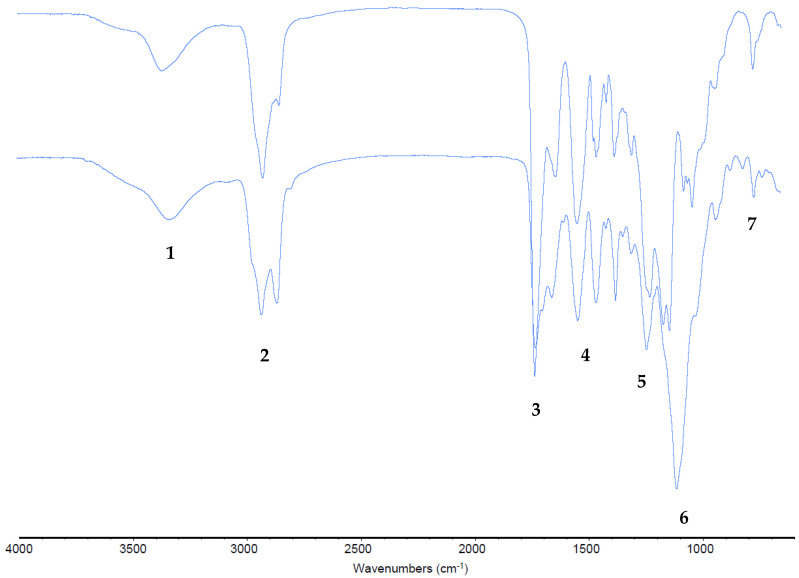
FTIR representative spectra, here shown in the case of sample 1, on the top outside finished layer and down the underside leather layer.

In the case of infrared spectroscopy, it was used for the identification of the polymeric layer. The finished layers of all three main samples and the separated pieces of sample 1 were analyzed (Figure 4); they belonged to the same polymer family: polyurethanes (PU). In fact, the substance that was made was possible to determine with a good approximation. This was conducted by analyzing the amount of infrared radiation absorbed, or transmitted, by a sample.

**Figure 5:**
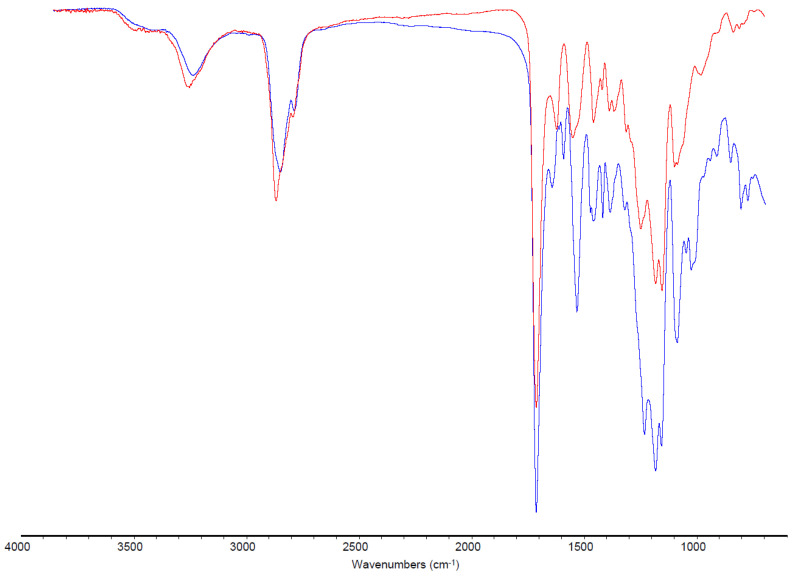
Uncolored layer (blue, down line) and a classic polyurethane (red, upper line) overlay.

There were some differences in substituents and quantities of CO and NH isocyanides. It confirmed the mixture of different polymers and the variety of them in similar samples. Where they were a mixture of several substances, the general characterization became more complex. Fortunately, the resulting spectra were very similar. In particular, at 3300–3500 cm^−1^ (1) there was the normal NH stretching of amines, at 2850–3000 cm^−1^ (2) there was the usual CH stretching of alkanes, at 1630–1750 cm^−1^ (3) there was the ordinary CO stretching of amides and esters, at 1500–1690 cm^−1^ (4) there was the classic NH scissoring of amines and amides, at 1350–1440 cm^−1^ (5) there was the usual CH deformation of alkanes, at 1000–1300 cm^−1^ (6) there was the common CO stretching of esters, at 1000–1250 cm^−1^ there was the ordinary CN stretching of amines, at 720–725 cm^−1^ there was CH rocking of alkanes, and at 660–900 cm^−1^ (7) there was the ordinary NH wagging of amines [37]. It has to be kept in mind that the 900–600 cm^−1^ area is the fingerprint, the characteristic area of every particular compound [38]. All these signals represented functional groups of polyurethanes. An overlapping of the polymer species spectrum with a general PU (aliphatic PU from amine-exposed polyester ATR ZnSe) in Figure 5 was also conducted to be sure in the case of an uncolored finished layer. It confirmed the polymeric main structure; therefore, these spectra were compared to find more characteristics of the analyzed polymers. An accurate spectra analysis, arrived at from a peak interpretation and a comparison with polymer libraries, reveals that the outside layer, colored and soluble in DMSO and DMF, was PU with aliphatic chains. The inside layer in front of leather, maybe the uncolored, insoluble, and shiny one, was mainly ether-based PU [39].

These results also confirmed that the polyurethane used is functionalized and different from the normal one, which could permit an explanation of why it was so difficult to dissolve these layers. Moreover, generally, in the finishing step, the leather surface changes some characteristics due to not only one product but many, and usually they are mixed products [40]. Another interesting confirmation is the complete absence of collagen [41]. It was an important result to exclude leather damage or cryogenic substrate removal. This was a further confirmation of the method’s effectiveness and validation.

### 3.4. SEM

Despite a direct visual analysis, it could be possible to see if leather turned crusty; for better comprehension, this kind of microscopy was conducted [42]. This analysis was conducted for a better visual of the top, bottom, and sides of the leather before and after the finished layer remotion. They reveal an important leather surface modification, a witness to a high change in the collagen fiber status. It permitted the observation of the collagen fiber disposition and confirmed the complete polymer removal (Table 3).

These fibers changed status from being more compact and tidier to being free and messy. Normally, with the polymeric layer, these are more ordered and bonded. If this part is removed, these become more free and superficially untied, ready for a new finishing treatment, perfectly achieving the objective of this innovative recycling method [43]. This could demonstrate complete polymer removal. In fact, the finished layer deposition is the step that could start to take the correct disposition, according to fashion and customer taste.

A possible problem could be the modification of all leather layers in terms of stability and collagen disposition, maybe for contact with very low temperatures. However, a reassurance about that arrived from SEM images “leather down,” in particular the collagen fibers not directly interested in remotion. It was permitted to demonstrate if there could be a leather modification after this treatment at −200 °C. With careful observation, collagen fibers stayed in a similar disposition and conformation without any particular changes.

So, these images demonstrated the innovative capacity of this new removal method and differentiated it from the other partial disruptive methods to recycle leather [44].

### 3.5. Tensile Strength according to UNI EN ISO 3376:2020

**Figure 6:**
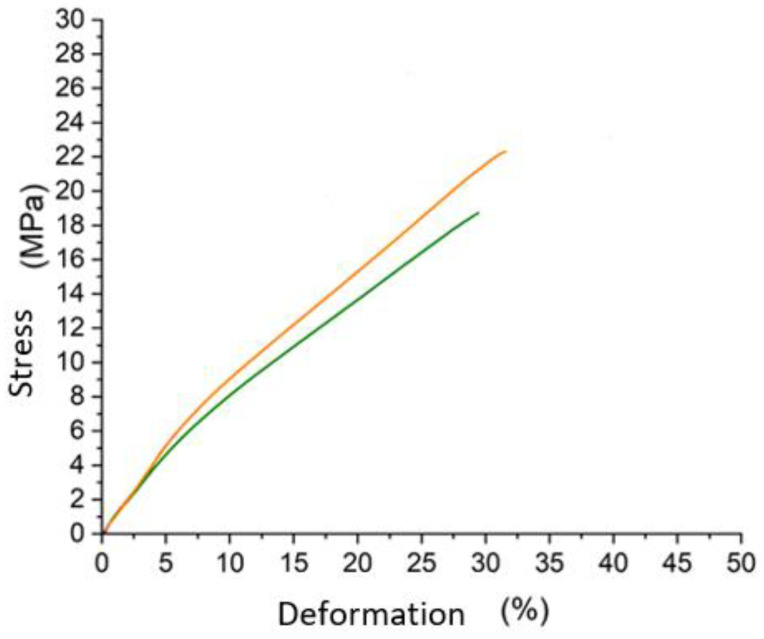
Stress/deformation chart obtained by tensile tests at 23 °C. As a representative case, it was used sample 2, green line with finished layer, and yellow line without the application of cryogenic method.

This normative was applied to check if there were differentiations in terms of qualities on leather and to be sure about the crust leather resistance after nitrogen treatment. Samples were compared with and without treatment, and generally, leather with a finished layer had more resistance than without. The polymers’ structure reinforcement takes the lead. At the same time, these results were expected to be really similar to the normal resistance values. So, it could be possible to demonstrate the good resistance of leather even after nitrogen treatment by the chart in Figure 6.

Surprisingly, leather with this treatment not only remained resistant; however, in one case over three, it improved its strength characteristics (Table 4) [45]. This is because, generally, the finishing operations are conducted to take advantage of some visual effects, touch effects, and permeation resistance [46]. It is important, and depending on which kind of polymers are applied, it could change lots of characteristic quantities. So, tensile resistance is not the main discriminative characteristic of this kind of leather. Or, to put it better, it already has a high resistance, and a little decrease does not pay much attention. It could be due to the collagen fibers’ compression and the lower elasticity of the polymers applied to this substrate. Anyway, these results were quite similar among the same sample; therefore, these were very positive answers for this new method. Interest was drawn to the brown sample (sample 2), which apparently was like sample 1; however, experimentally, it had a stronger resistance and a different profile. Before, there was a high stress resistance, and after that, the slop was less, remaining high. Another way to see these important results is in Table 4.

These results opened doors to the possible real affirmation of this new method in terms of performances and results. In fact, the products had very high performances, which made them the best substrate for another finishing step.

### 3.6. DSC

**Figure 7:**
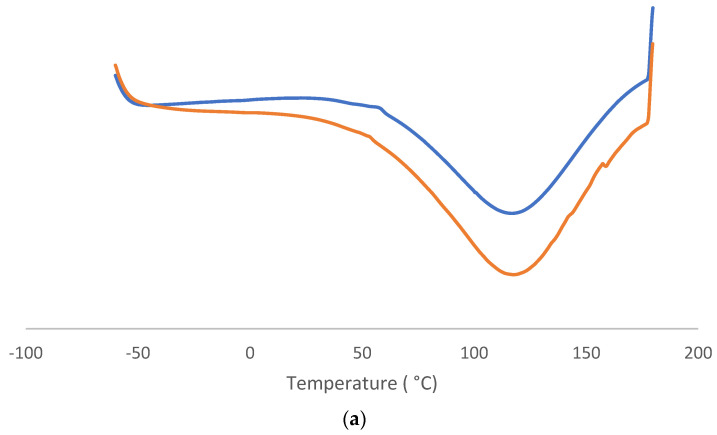

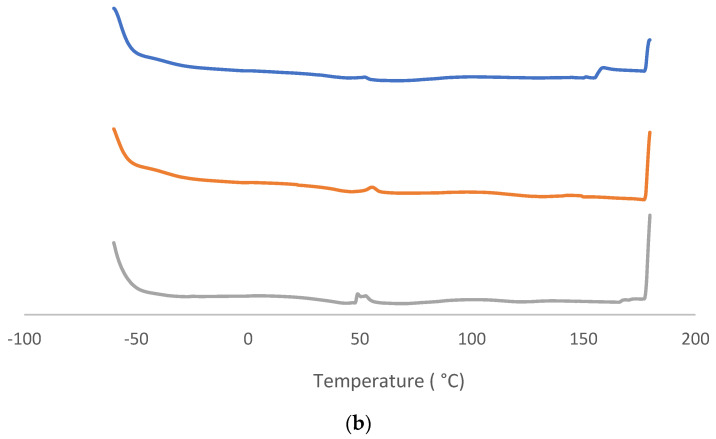
DSC charts (temperature vs. heat flow): (**a**) leather thermograms, from below: orange line was for sample 1 without finished layer and blue line was for blank sample 1; (**b**) it represented the overlay of all finished layers, from below: grey line for light brown (from sample 3), orange line for black (from sample 1), and blue line for brown (from sample 2).

This kind of analysis was used to characterize and gain a better understanding of the thermal behavior of leather after the nitrogen method and polymeric layer [47]. It represented if there was differentiation in terms of exo and endothermic transformation between −60 and 180 °C. To be sure of that, two complete cycles (from cold to hot and from hot to cold) were conducted. It could confirm the results and say what the materials’ attitude was. The thermograms were grouped in terms of samples, and charts were conducted such as heat flow based on temperature or time. In this article, the black sample thermogram (Figure 7) is representative of the other samples (available on Supplementary Materials).

From leather graphs, the first interesting phenomenon was the great endothermic curve during the first heat cycle. It seems to correspond to water evaporation, an endothermal transformation that occurs after the first cycle. Anyway, it is much more possible to have a glass transition [48], which is also assessable in four-cycle charts for the partial reversibility of collagen; for this reason, the third cycle was different from the first one. The profile was similar for all the kinds of leather, with a marked difference only in the relaxing trend. It was interesting to see the exothermic curve at 60–80 °C, maybe due to a possible mechanism that could happen only in the case of leather plus polymer. The comparison of leather with and without a finished layer represented the protection polymer property to the heat flux. It was useful for a better determination of the sample’s characteristics. At the same time, the changes in them after and before nitrogen generally respected the normal thermal behavior without such strong differences. It was a further confirmation of the high qualities remaining inside treated leather. In b it was possible to see a regular thermal profile without any great changes. The more defined and regular curve demonstrated a similar physical status of all PU on leather and similarity in thermal characteristics in this range of temperatures. The first endothermal curve that represented the glass transition was around −50 °C, and the first exothermical curve that represented the crystallization process was around 50 °C [49]. Anyway, the substantial difference between b and the others attested another time to the absence of leather in the finished layer.

### 3.7. TGA-TMA

**Figure 8:**
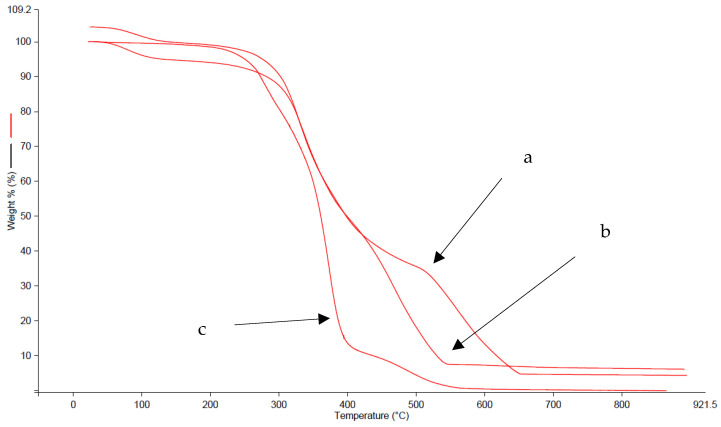
(**a**) blank test sample 1; (**b**) leather of sample 1; (**c**) finished layer of sample 1.

This analysis was conducted for a comparison with DSC, to know what happened at high temperatures on substrate samples, and to check if it was possible to perform a weight loss analysis [50]. The behavior was studied between 30 and 900 °C. It permitted a complete overview of all the main characteristics of these materials as well as a better understanding of their polymeric role [51]. The results could be well explained by sample 1 thermogram (Figure 8).

It was possible to see similar transition peaks and weight loss ranges in a and b. At the same time, there were high differences in c, with one main transition in a short temperature range. Unfortunately, the decomposition of polymers and leather started at the same temperature. For this reason, it was not possible to separate and quantify them by isotherm. Another interesting aspect was the protection of leather by this polymeric layer at high temperatures; in fact, all three samples represented this situation. A particular characteristic, such as on tensile strength analysis, is the high resistance of sample 2. It was not due only to mechanical properties but also to thermal qualities. Some phenomena occurred: in the case of leather, the remaining weight is the inorganic part, chromium mainly (judging by the color). Another is the indirect leather weight diminution, in line with the DSC behavior, to attest to the undefined and different status of collagenic structures. Similar was the case with polymers. Another result that confirmed the DSC discussions was the major temperature resistance of blank-protected leather [52]. Polymers had a similar behavior; they started a fast decomposition. For the cryogenic delamination method, TGA-TMA represented the possibility of attesting the absence of leather in polymer, as in DSC. It was a useful analysis for a comparison of these techniques, for a thermal result validation, and to have a complete idea of thermal behavior before and after nitrogen.

Through all these analyses, it was possible to check the potentialities of the nitrogen-finished layer removal method. It has to be better developed; however, these data encourage this new principle, which could change the recycling field. Costs are the limiting factor; however, with the unique versatility and the improved leather value, it will be possible to seize this important opportunity.

## 4. Conclusions

The global population grows every day; however, the world has the same area. So, the only possibility to survive in good condition without a marked footprint—the chance to develop and improve human life—is a sustainable approach. It must pay attention to a lot of different things that, years ago, were not focalized. It has to improve the value of many things that, for years and years before, were only considered waste. However, this is the better way to walk, for continuing to live together, according to the Sustainable Development Goals (SDGs) of the United Nations (UN). In this scenario, the article indicates one possible solution to take value from or improve the value of the leather scraps. It could revolutionize the recycling methods for the leather sector through the complete use of collagen without a great loss of substrate, energy, or chemicals. It represents a strong alternative possibility to shaving or hydrolyzing (both techniques involve a loss of leather). Maybe it could be seen as something not so important compared to the huge problems of environmental change, carbon footprint, etc. However, all is connected, and a reinforcement of the circular economy in the leather industry could become an important brick for all other steps toward improving the sustainability of this sector. The use of liquid nitrogen for scrap revaluation is a surprising solution, and the primary resource could become easy to restore in a closed system. Moreover, the machines that will use this technology will work continuously and in common, such as one per district. This approach must be placed in the context of the liquid nitrogen engine revolution, with all the challenges and developments in the future.

## 5. Patents

P07501/IT.

## Figures and Tables

**Table 1:** This table reports the main general characteristics of leather samples. For the operative conditions, check the next paragraph, “cryogenic delamination”.

Sample	Thickness before (b) and after (a) (mm)	Look	Color	Photo before	Photo after
Entry 1	1.64 (b) 1.54 (a)	Full hand	black	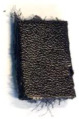	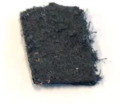
Entry 2	1.56 (b) 1.36 (a)	Compact touch	brown	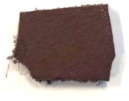	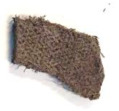
Entry 3	1.29 (b) 1.11 (a)	Soft hand	Light brown	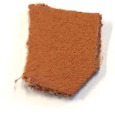	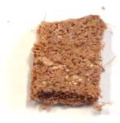

**Table 2:** Solubility in different solvents of several samples at room temperature.

Sample	Solvent		Dissolution Activity
	Name	Structure	
Sample 1 finished layer	Distilled water		Negative
	DMSO	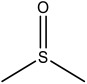	Partially
	THF	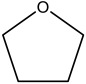	Negative
	Chloroform	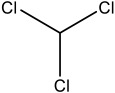	Polymer separation
	Acetonitrile	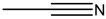	Negative
	Ethanol	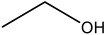	Negative
	n-hexane	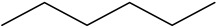	Negative
	DCM	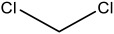	Negative
	Cyclohexanone	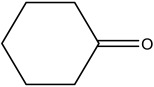	Polymer separation
	Acetone	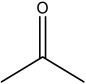	Negative
	Methyl tert-butyl ether	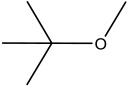	Negative
	Toluene	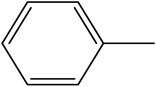	Negative
	Butanone	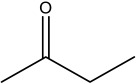	Negative
	Methyl acetate	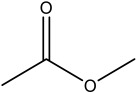	Negative
	Ethylene glycol	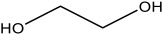	Negative
	DMS	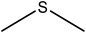	Negative
	Dioxane	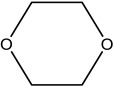	Negative
	DMF	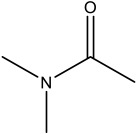	Partially
Sample 1 uncolored finished layer	THF		Negative
	Acetone		Negative
	Acetonitrile		Negative
	Methanol	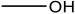	Negative
	Distiller water		Negative
	Toluene		Negative
	DCM		Negative
	DMF (100 °C)		Negative
	Dioxane		Negative
Sample 2 finished layer	DMSO		Partially
Sample 3 finished layer	DMSO		Negative

**Table 3:** SEM images, before and after nitrogen treatment, collagen status.

Before	After
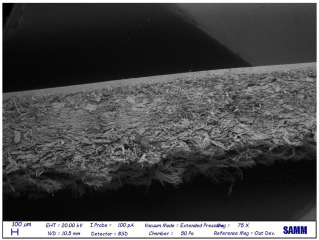	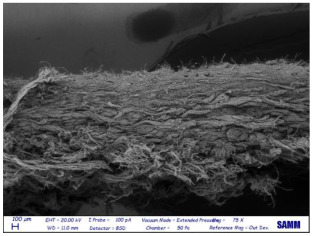
Leather side	
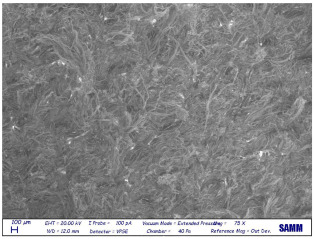	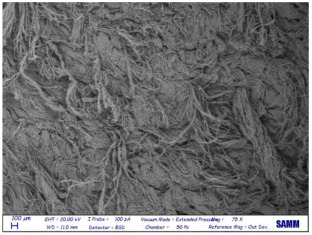
Leather top	
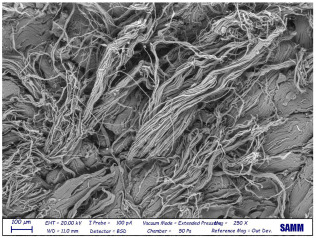	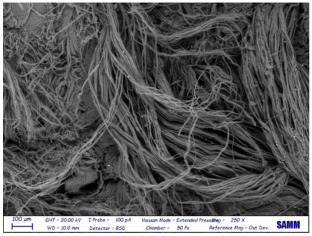
Leather down	

**Table 4:** Main values evaluated on tensile strength assay; the entries represented 1: blank sample 1; 2: sample 1 with cryogenic method; 3: blank sample 2; 4: sample 2 with cryogenic method; 5: blank sample 3; 6: sample 3 with cryogenic method (with cryogenic method means without finished layer).

Entry	Deformation at Maximum Stress (%)	Maximum Stress (MPa)
1	36.7	20.6
2	40.4	23.4
3	31.6	22.3
4	29.8	18.7
5	35.1	22.3
6	34.1	20.4

## Data Availability

Not applicable.

## Supplementary Materials

The following supporting information can be downloaded at: https://www.mdpi.com/article/10.3390/ma16186166/s1, Figure S1: DSC chart of sample 3 finished layer; Figure S2: DSC chart of sample 1 finished layer; Figure S3: DSC chart of sample 2 finished layer; Figure S4: DSC chart of sample 1 without finished layer; and Figure S5: DSC chart of blank sample 1.
