# Supplementary material for: A Circular Approach to Finished Tanned Leather: Regeneration by Cryogenic Technology
Source: Materials (Basel). 2023 Sep 11;16(18):6166. doi: 10.3390/ma16186166 (PMC10533182; doi:10.3390/ma16186166)
Supplement: Supplementary file 1 [file materials-16-06166-s001.zip › materials-2571012-supplementary.pdf]

materials

Supplementary Materials

A Circular Approach to Finished Tanned Leather: Regeneration by Cryogenic Technology

Omar Salmi, Simone Gelosa, Filippo Rossi and Maurizio Masi *

DSC charts with heat flow

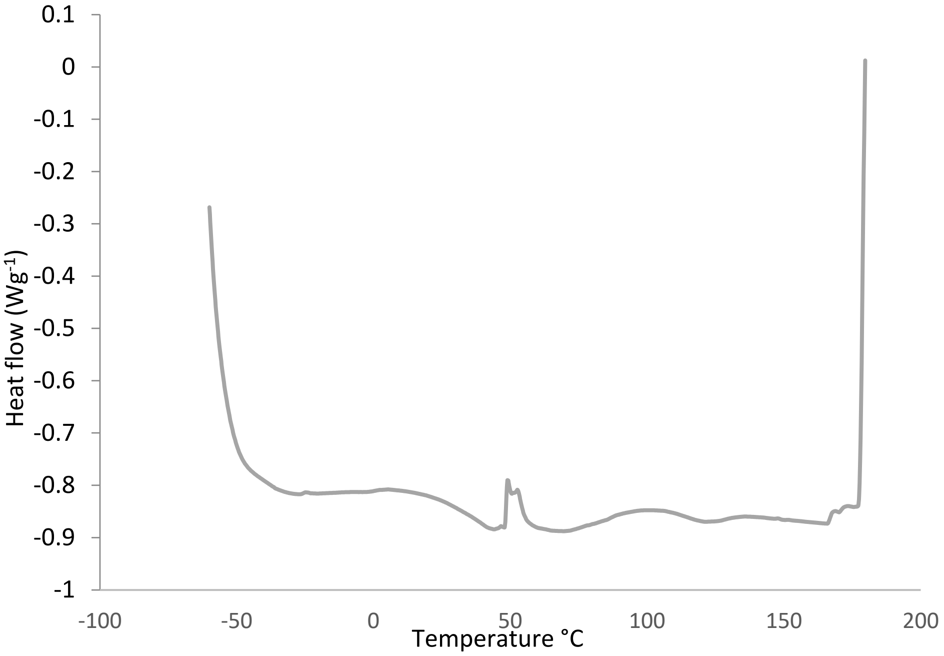

Figure S1: DSC chart of sample 3 finished layer.

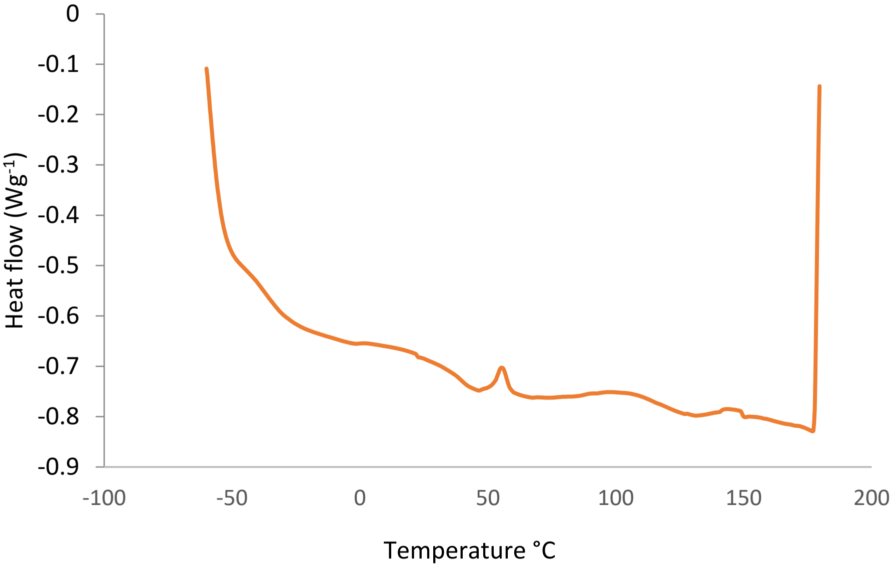

Figure S2: DSC chart of sample 1 finished layer.

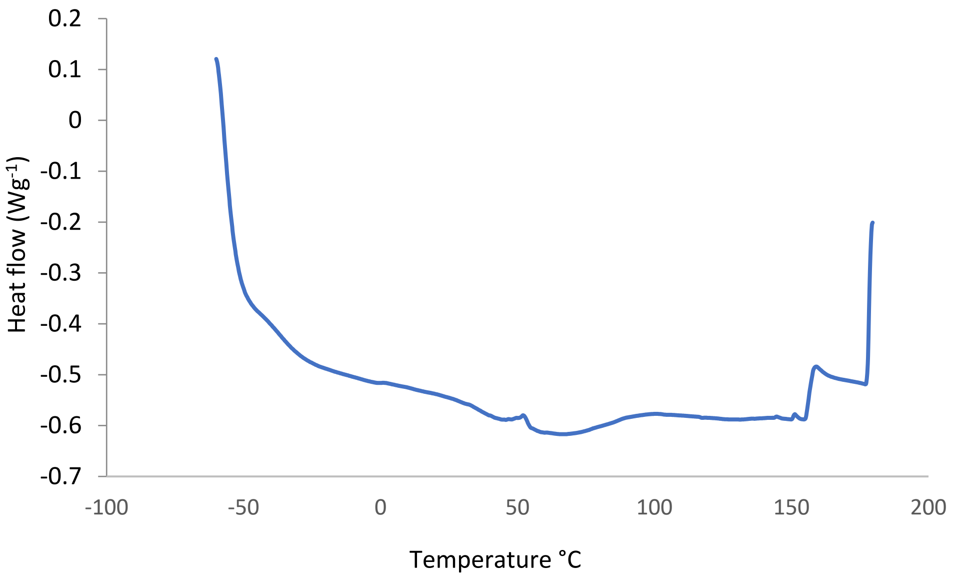

Figure S3: DSC chart of sample 2 finished layer.

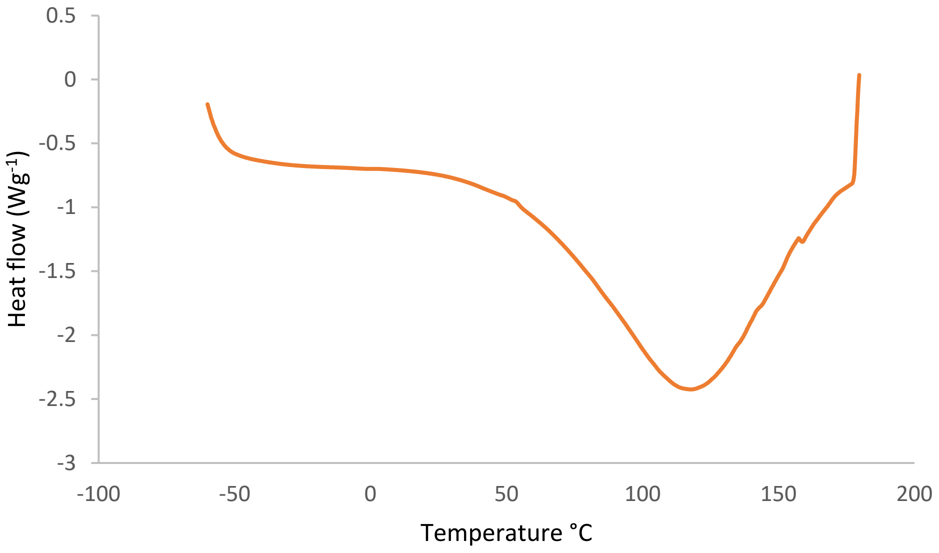

Figure S4: DSC chart of sample 1 without finished layer.

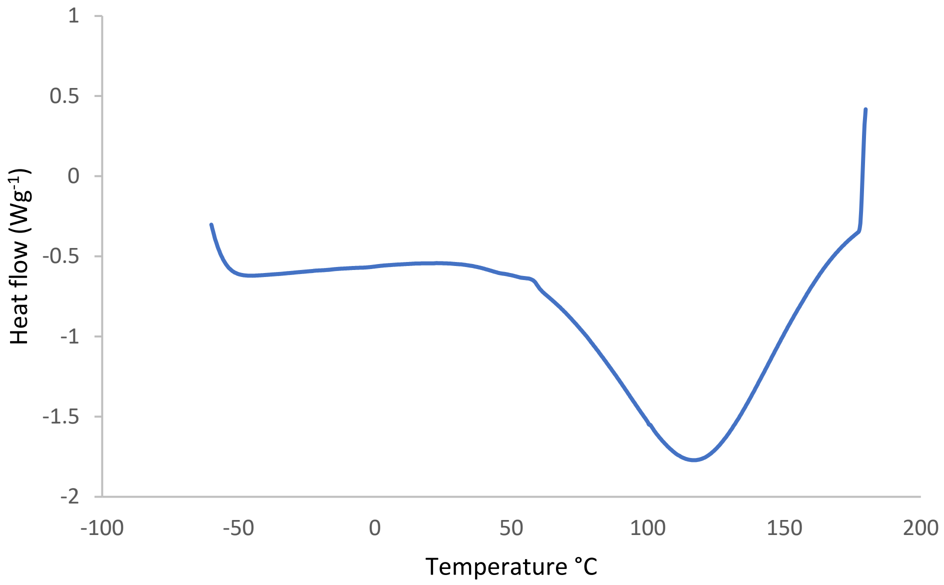

Figure S5: DSC chart of blank sample 1
